# A pilot longitudinal study of decrease in cognitive functions during the most painful day of the period among women with primary dysmenorrhea

**DOI:** 10.1007/s00404-024-07617-9

**Published:** 2024-07-04

**Authors:** Julia Kluska, Ewa Malinowska, Joachim Kowalski

**Affiliations:** 1 Faculty of Psychology, University of Warsaw, Warsaw, Poland; 2 Faculty of Psychology, University of Warsaw, Warsaw, Poland; 3 Experimental Psychopathology Lab, Institute of Psychology, Polish Academy of Sciences, Warsaw, Poland

**Keywords:** Menstrual pain, Cognitive assessment, Attention, Cognitive control

## Abstract

**Purpose:**

Painful menstruation is a common problem associated with many limitations in day-to-day functioning. There is limited research on the temporary effects of menstrual pain on cognitive functioning.

**Methods:**

A longitudinal pilot study was conducted. A group of 32 women was tested with the Brief Test of Adult Cognition by Telephone (BTACT), which consists of 7 tests measuring various cognitive functions. Participants were tested both on a regular, pain-free day and the most painful day of their period.

**Results:**

The subjects displayed significantly lower results in several domains of cognitive functions during measurement on the most painful day of the period. We observed a decline in inhibitory and cognitive control, attention functioning and processing speed.

**Conclusions:**

The results provide tentative evidence for experiencing cognitive difficulties under the influence of menstrual pain and may contribute to raising awareness about related difficulties.

## What does this study add to the clinical work

**Table:** 

The study highlights the significant short-term impact of menstural pain on cognitive functioning,specifically in inhibitory and cognitive control, attention and speed processing, a previously not studied in a longitudinal form. The findings emphasize the importance of addressing menstural pain as a potential contributor to short-term cognitive difficulties.	

## Introduction

Dysmenorrhea is considered one of the most frequent gynecologic disorders, with over 50% of post-pubescent women experiencing painful menstruations [3]. We can distinguish between two forms of it: primary, which is characterized by menstrual pain without any noticeable pelvic lesions, and secondary, where painful menstrual bleeding is associated with specific disorders, such as endometriosis [17]. Primary painful menstruation differs from the frequently experienced menstrual cramps in the need for treatment, usually because the woman is unable to perform usual activities [16]. Primary dysmenorrhea can be considered an umbrella term, applying to any person without visible pelvic lesions who experiences disruptive menstrual pain. Despite some known risk factors like nulliparity, heavy menstrual flow, and smoking, a cause of this disorder has still not been established, besides specifying the release of prostaglandins in the menstrual fluid as the main pathogenic correlate [18]. This phenomenon, in addition to purely medical symptoms, often has psychological consequences. Previous studies have shown an overall lower quality of life index in women with dysmenorrhea compared to healthy ones, and this relationship was stronger the more severe the pain was declared by the subjects [2, 20]. A Canadian study [19] showed that 60% of women over the age of 18 experiencing one form of the syndrome declared experiencing severe or at least moderate pain, while 51% of these subjects declared limitations in various daily activities, 17% of whom missed work or school due to the discomfort in question. Due to the limitations caused by menstrual pain, people experiencing it have to give up many activities, including participation in school or work life. The cited studies considered the overall level of quality of life in the subjects, but Iacovides and her team (2013) decided to investigate the relationship in more depth, taking into account the phase of the cycle. In a longitudinal study, it was shown that women experiencing painful menstruation declared a significant deterioration in quality of life during the first day of the menstrual cycle compared to the control group. These data allow us to conclude that at the moment of experiencing pain people with dysmenorrhea feel most restricted, resulting in exclusion from many activities and reduced quality of life. In a meta-analysis by Bajalan et al. [15], women experiencing primary dysmenorrhea showed the prevalence of anxiety and depressive disorders significantly higher than in healthy subjects, and there also was a correlation of primary dysmenorrhea with experiencing stress. A relation between experiencing menstrual pain and elevated anxiety and depression symptoms was recorded by [11]. Weakened social support observed in the declarations of women experiencing painful menstruation may also be a correlate of the deterioration in quality of life and an increased susceptibility to mental disorders [28].

Taking into account the positive association between experiencing general pain and a brief worsening of cognitive functions (Moriarty, McGuire, Finn, 2011), experiencing dysmenorrhea could result in temporary decrease in cognitive functions. There are several studies [8, 9] which point out that dysmenorrhea may cause some adverse general functioning effects. These studies demonstrated that during the period phase of the menstrual cycle, attention processes are slowed down and more errors are being made by the subjects. However, data on cognitive functioning in people experiencing primary dysmenorrhea are still scarce. Most studies researching cognitive aspects of dysmenorrhea are cross-sectional and compare people with and without symptoms and do not control for the stage of the menstrual cycle [1, 6]. Despite that, experiencing menstrual pain should not be perceived only as a person’s quality which lowers their overall cognitive performance, but also as a brief state which impairs cognitive functions temporarily.

What is more, qualitative studies have found that people with primary dysmenorrhea are in a constant "state of alert," as they require much more frequent access to the restrooms or need access to sanitary products and medications [23]. In some cases, surveyed young women did participate in school activities despite the pain, but they declared problems with concentration and an inability to remember school material (Fernández-Martínez et al., 2020b).

As there are few studies on the subject, the aim of this pilot study was to explore the impact of menstrual pain on the level of cognitive functions among people experiencing primary dysmenorrhea in a longitudinal design. Basing on previous studies on pain and cognitive functions (Moriarty, McGuire, Finn, 2011) and on the relation between menstrual pain and attention [9], it was hypothesized that different domains of cognitive functions (working memory, attention, cognitive control, inhibitory control, reaction time, speed of processing) will be lower during a painful day of the menstrual cycle than its level on a painless day among women suffering from primary dysmenorrhea.

## Methods

### Procedure and Sample Selection

This work was supported by funds for the development of research potential of the Faculty of Psychology at the University of Warsaw granted by the Ministry of Science and Education (501-D125-01–1250000 commission. 5,011,000,236). The study was conducted between December 2020 and February 2021 with at least a week between measurements. Study took place during the peak of COVID-19 pandemic, so the participants were qualified through an online survey, and the study was conducted through telephone in order to maintain social distance. The study was approved by the Ethics Committee of the Faculty of Psychology, University of Warsaw.

The subjects were recruited from groups concerning women’s health on Facebook. The main requirement to be qualified for the study was to experience primary dysmenorrhea, which was established through self-reports. To meet this criterion, subjects had to declare experiencing at least one symptom from each of two proposed categories of symptoms associated with painful menstruation: pain symptoms (abdominal pain, visceral cramps in the lower abdomen, hip pain, lower back pain, pain on the inside of the thighs) and symptoms secondary to pain (nausea and vomiting, diarrhea, fatigue or fainting, difficulty concentrating) [3, 13] and have no comorbidities, including diseases or suspected diseases of the pelvic area. The self-report technique was used due to the online nature of the study.

Measurements were conducted on two separate days. The first measurement was conducted on a painless and painkiller-free day, and the second measurement on the first or second day of the menstruation, depending on when the subject declared subjectively experiencing most significant pain. One measurement was conducted in reverse order upon request of the participant. Subjects were asked not to take any painkillers during the measurement days in order not to confound the results. It was originally intended that the order of measurements would be randomized to control for effects of being familiar with the tasks and learning, but people who had their first measurement on a painful day gave up on the follow-up measurement, so this intention was eventually abandoned.

The preliminary survey was completed by 241 people of which 55 met the criteria of primary dysmenorrhea. None of the participants had a diagnosis or suspicion of any lesions to pelvic regions, thus secondary dysmenorrhea diagnosis was rejected. Finally, n = 32 participants took part in both parts of the study and these results were analyzed. Most of the subjects were in their twenties (*M* = 23.94, *SD* = 6.73), all were women, most experienced menstrual pain every cycle (46.9%) or almost every cycle (40.6%) and declared abdominal pain (96.9%), visceral cramps (87.5%), diarrhea (75%), fatigue or fainting (71.9%) lower back pain (62.5%) and difficulty concentrating (56,3%) as the most common accompanying symptoms, 59% declared experiencing over 5 out of 9 possible symptoms.

### Measures

Cognitive functions were measured with scores on individual tasks from the Brief Test of Adult Cognition by Telephone (BTACT) [10] in two versions—C and D, assigned to participants in a counterbalanced order. The battery consisted of 7 subtests—Rey 15-item Test (direct and delayed), Backwards Digit Span, Verbal Fluency Test, Go/No Go Test, Number Sequence Test, Backwards Counting Test. Higher scores indicate better cognitive performance (apart from the Backwards Counting Test, in which the lower the raw score the better the performance). The tool is considered an efficient and effective method of testing cognitive ability among healthy non-clinical adult population in all age groups and from a range of educational background [14]. Despite the tool's option to calculate a component score, this study opted to use each task as a separate indicator of a specific cognitive function, rather than obtaining a general cognitive functioning score.

### Statistical analyses

To determine the sample size needed to reliably detect effects of interest, we conducted a power analysis with G*power software [5]. To reliably detect a medium effect size (d = 0.5) with β = 0.80 and one-tail α = 0.05 (given the directional nature of our hypothesis), a sample size of *n* = 27 participants is needed.

Repeated measurements of tests of cognitive functions were analyzed with a paired *t* test. If normality of distribution assumption was not met (significant result of a Shapiro–Wilk’s test), Wilcoxon ranked test was used. Cohen’s d was used to estimate effect sizes with Psychometrica software [22].

## Results

Analyses of tasks performances showed that the mean raw scores in: Word Fluency Test, Go/noGo Test, Backwards Counting Test on the non-painful day were statistically significantly higher than the mean scores in these tasks on the painful day. Similar analyses of the remaining tests (direct Rey's 15-item Test, delayed Rey's 15-item Test, Backwards Digit Span) showed no statistically significant differences in the mean scores of the tasks on the non-painful day compared to the painful day. Detailed results of the tasks and analyses are shown in Table 1.

**Table 1:** Mean final raw score in the tasks for painful and non-painful day and the value of the Student's *t* test or Wilcoxon's ranked signs for the analyzed mean difference in measurements

Test	Cognitive functioning domain	Raw results (non-painful day)		Raw results (painful day)		*t* test or Wilcoxon’s W	Cohen’s d
		M	SD	M	SD		
Direct Rey 15-item test	Episodic verbal memory	10.06	2.50	8.90	2.48	−1.43	−0.25
Backwards digit span	Working memory span	4.47	1.16	4.22	1.32	1.03	0.18
Verbal fluency test	Executive function (cognitive control), semantic memory and speed of processing	29.94	6.42	27.50	5.92	2.20*	0.39
Go/noGo	Reaction time, attention, task switching, inhibitory control	67.03	3.34	59.03	5.67	6.79**	1.2
Delayed Rey 15-item test	Episodic verbal memory	10.59	2.01	8.97	2.26	−0.15	−0.03
Number sequence	Fluid intelligence	2.38	1.54	2.5	1.41	111.5	−0.10
Backwards counting test	Processing speed, cognitive control	68.19	7.47	70.66	6.86	60.5**	−0.65

*M* mean; *SD* standard deviation. * *p* < 0,01; **p < 0,001

Analyses of errors in the tasks showed a statistically significantly lower number of interjections on the Rey 15-item Test on the non-painful day than on the painful day. This relationship was found in the direct and the delayed condition of the test. For the remaining errors (number of repetitions in the Rey 15-item Test in the direct and delayed condition, number of repetitions in the Word Fluency Test, number of errors in the Backwards Counting Test), no statistically significant differences were shown between the non-painful and painful day. The detailed results of the correlations for susceptibility to errors are shown in Table 2.

**Table 2:** Average results for errors tendency in the tasks for painful and non-painful day and the value of the Wilcoxon ranked signs for the analyzed difference in mean measurements

Test	Type of error	Non-painful day		Painful day		*Wilcoxon’s W*	*Cohen’s d*
		*M*	*SD*	*M*	*SD*		
Direct rey 15-item test	Repetitions	0.31	0.53	0.13	0.34	1.89	−0.4
	Inclusions	0.5	0.62	0.75	0.80	2.14*	0.35
Verbal fluency test	Repetitions	0.53	0.88	0.72	1.02	0.91	0.2
Backwards counting test	Sum of counting errors (additions, repetitions and omissions)	0.28	0.52	0.41	0.71	0.71	0.21
Delayed rey 15-item test	Repetitions	0.43	0.72	0.28	0.63	1.13	−0.22
	Inclusions	0.63	0.71	0.94	0.88	2.13*	0.39

*M* mean, *SD* standard deviation. * *p* < 0,01; **p < 0,001

## Discussion

This study explored the short-term impact of menstrual pain on cognitive functioning. The observed deterioration in the processes studied is limited in time only to days when severe menstrual pain was experienced. The obtained results partially confirm the assumed hypothesis regarding the worse cognitive functioning during a painful day of menstruation compared to a painless day. We observed a deterioration of attention, as well as cognitive and inhibitory control, reaction time and speed of processing and task switching, but no significant impairment of working and episodic verbal memory. These findings are generally in line with previous study on longitudinal effects of menstrual pain on attentional processes [9]. This pattern of results could be explained by two potential mechanisms: either the skills tested are so basic and automatic that they are not affected by pain, and the slower pace is due to a generally slowed processing and reduced attention span [12, 25]; Bosma & Kessels, 2020), or alternatively, pain enhances the attentional processing load, forcing longer verification of responses before uttering them, leading to longer reaction times—an example of a compensation mechanism [27]. Both of these explanations point to a common element: a greater load on cognitive processes during pain, which interferes with their proper functioning.

These results suggest relationships between experiencing pain and lower psychosocial functioning [2] in primary dysmenorrhea may be at least partially explained by decreased cognitive functioning during certain phases of the menstrual cycle. Prolonged periods of pain and physical discomfort adversely affect cognitive capacity. It may be further hypothesized that such experiences put strain on daily functioning of people affected by dysmenorrhea, and are fostered by stress, adverse emotional experiences and symptoms of depressive–anxiety symptoms (e.g., [7]).

Our study has some limitations. In our study, the inclusion criterion for 'primary dysmenorrhea' was based on self-reported information from the subjects rather than clinically established diagnosis, which may introduce a degree of subjectivity and potential bias that should be considered when interpreting the results. It is worth mentioning that in reality studied effects could be of greater size, but due to the learning effect present during this study, the observed effects are underestimated. This effect was partially mitigated using different versions of BTACT. However, future studies should aim to counterbalance the order of measurements. The study sample proved to be too small to distinguish between users and non-users of hormonal contraception. The majority of the people surveyed were women aged 20–23, so the correlations obtained in the study should be regarded as characteristic of people in the aforementioned age group. In order to be able to generalize the results to the entire population, a similar study should be carried out on a sample with a larger representation of people aged over 30.

A notable limitation of our study is the potential influence of the hormonal cycle on cognitive function [4], which could confound the observed effects attributed to pain. While this does not undermine the role of pain, it is important to consider that pain perception might be modulated by hormonal fluctuations [29]. Additionally, mood is a potential confounding variable that was not measured in this study, especially during the pandemic period (Helbig, et al. 2023). Negative mood induction in chronic pain patients increases self-reported pain, indicating its role in pain perception [31]. Future research should employ questionnaires to concurrently assess pain and mood, allowing for a joint regression analysis to discern the relative contributions of these variables to cognitive function. Such an approach would enhance our understanding of the physical–mental interplay, as posited by the bio-psycho-social model.

In future, it would be worthwhile to aim to compare cognitive functioning of individuals with and without dysmenorrhea in different phases of the menstrual cycle and use a larger study sample that would allow analysis of the study relationship by subgroups in terms of contraceptive use.

## Conclusions

The observed results tentatively point to the difficulties of young women with primary dysmenorrhea and highlight the impact of pain on basic cognitive functioning, especially domains of attention and executive functioning. The study is a step toward a better understanding of the phenomenon of painful menstruation and related difficulties.

## Data Availability

The obtained data have been uploaded to the manuscript as supplementary material.
